# The Positional Isomeric Effect on the Structural Diversity of Cd(II) Coordination Polymers, Using Flexible Positional Isomeric Ligands Containing Pyridyl, Triazole, and Carboxylate Fragments

**DOI:** 10.3390/molecules23102634

**Published:** 2018-10-14

**Authors:** Jonathan Cisterna, Catherine Araneda, Pilar Narea, Alejandro Cárdenas, Jaime Llanos, Iván Brito

**Affiliations:** 1 Departamento de Química, Facultad de Ciencias Básicas, Universidad de Antofagasta, Casilla 170, Antofagasta 1240000, Chile; jonathan.cisterna@uantof.cl (J.C.); catherine.araneda@gmail.com (C.A.); pilar.narea@uantof.cl (P.N.); 2 Departamento de Física, Facultad de Ciencias Básicas, Universidad de Antofagasta, Casilla 170, Antofagasta 1240000, Chile; alejandro.cardenas@uantof.cl; 3 Departamento de Química, Universidad Católica del Norte, Av. Angamos 0610, Antofagasta 1240000, Chile; jllanos@ucn.cl

**Keywords:** metal-organic frameworks, Cd(II) complexes, X-ray diffraction analysis, thermal properties, luminescence

## Abstract

To systematically investigate the influence of the positional isomeric effect on the structures of polymer complexes, we prepared two new polymers containing the two positional isomers ethyl 5-methyl-1-(pyridin-3-yl)-1*H*-1,2,3-triazole-3-carboxylate (**L1**) and ethyl-5-methyl-1-(pyridin-3-yl)-1*H*-1,2,3-triazole-4-carboxylate (**L2**), as well as Cd(II) ions. The structures of the metal–organic frameworks were determined by a single crystal XRD analysis. The compound [Cd(L1)_2_·4H_2_O] (**1**), is a hydrogen bond-induced coordination polymer, whereas the compound [Cd(L2)_4_·5H_2_O]_n_ (**2**) is a three-dimensional (3-D) coordination polymer. Their structures and properties are tuned by the variable N-donor positions of the ligand isomers. This work indicates that the isomeric effect of the ligand isomers plays an important role in the construction of the Cd(II) complexes. In addition, the thermal and luminescent properties are reported in detail.

## 1. Introduction

The self-assembly of coordination polymers and metal–organic frameworks (MOFs) [1,2,3] has been attracting great attention in the past decade, mainly because of their great potential as functional materials for diverse technological applications [4,5,6,7,8]. In particular, the luminescent properties of this type of material, as well as the possibility of fine-tuning the characteristics of their emission by carefully selecting both the metal and the organic ligands, have been a topic of special relevance in this field [9,10,11,12,13].

In this sense, the well-studied luminescent properties [14,15,16,17,18] of *d*^10^ cations, such as Cu(I), Ag(I), and Au(I), as well as their versatility in the construction of complex coordination networks with different types of organic ligands have been the object of interest.

From a structural point of view, the two principal themes in this field have been the synthesis of compounds that have either discrete molecular architectures with polyhedral or polygonal shapes or infinite coordination polymers in one, two, or three dimensions (1-D, 2-D, or 3-D, respectively) composed of metal ions in combination with deliberately tailored organic ligands. In the latter case, the resulting network topology [19] for the supramolecular complex can usually be predicted by selecting the chemical structure of the organic ligands and the usual coordination geometry of the metal ions linking the ligands together in the final structure [20]. The structural properties of the bridging ligands, such as their rigidity or flexibility, length, size, bulkiness, and linear or nonlinear geometry, have been found to play an important role in the construction of specific macromolecular architectures [21,22,23].

One crucial aim of this work is to explore the essential factors of positional isomeric ligands for regulating the structural assembly of Cd(II) MOFs, which may provide further insight into the design of new functional crystalline materials [24,25,26,27,28].

## 2. Materials and Methods

### 2.1. Reagents and Instruments

All chemicals were of A.R. grade and used without further purification (Sigma-Aldrich, St. Louis, MO, United States). FT-IR spectra in the range of 400–4000 cm^−1^ were obtained, using KBr pellets, with a Nicolet Avatar 330 spectrometer (Thermo Scientific, Waltman, MA, United States). The elemental analyses were obtained on a CNHS FLASH EA 1112 Elemental analyzer (Thermo Scientific, Waltman, MA, United States). HR-ESI-MS were obtained on a Waters (Micromass) AutoSpec mass spectrometer (Water co.; Milford, MA, United States). Powder X-ray diffraction data were obtained on a Bruker D8 Advance diffractometer (Cu-Kα radiation (λ = 0.1542 nm); Bruker Co.; Billerica, MA, United States). The thermogravimetric analyses were carried out in an N_2_ atmosphere on a Mettler Toledo DL31 thermoanalyzer (Mettler Toledo enterprise, Columbus, OH, United States) with a heating rate of 10 °C/min. The luminescence spectra were recorded on a JASCO FP-8500 spectrofluorometer (JASCO Co.; Kyoto, Japan). The excitation was performed with λ_ex_ = 360–370 nm, and the emission was recorded at λ_em_ = 410–450 nm.

### 2.2. Single-Crystal X-ray Diffraction

The diffraction data for compound **1** were collected on an automated D8 Venture Bruker diffractometer (Bruker Co.; Billerica, MA, United States) equipped with a two-dimensional CMOS detector (graphite monochromator, λ(MoKα) = 0.71073 Å, ω-scans). For compound **2**, λ(CuKα) = 1.5418 Å radiation (ω-scans) was used. Integration, absorption, correction, and determination of unit cell parameters were performed using the APEX3 program package [29]. The structures were solved by a dual space algorithm (SHELXT [30]) and refined by the full-matrix least squares technique (SHELXL [31]) in the anisotropic approximation (except hydrogen atoms). The final formula of compound **2** was calculated from the data of the PLATON/SQUEEZE procedure [32] (196 ē in 699 Å^3^, equivalent to around 10 disordered ethanol molecules). Additional crystallographic details are available in the CIF files. ORTEP views were drawn using OLEX^2^ software (version 1.12, Olexsys Ltd., Durham University, Durham, UK) [33]. The crystallographic data and details of the structure refinements are summarized in Table 1. CCDC 1515697 (**L1H**), 1515698 (**L2H**), 1866538 (**1**), and 1866353 (**2**) contain the supplementary crystallographic data for this paper. These data can be obtained free of charge from the Cambridge Crystallographic Data Center at http://www.ccdc.cam.ac.uk/data_request/cif.

### 2.3. Synthetic Procedures

#### 2.3.1. General Procedure for the Syntheses of Ligands **L1** and **L2**

The organic ligands ethyl 5-methyl-1-(pyridin-3-yl)-1*H*-1,2,3-triazole-3-carboxylate (**L1**) and ethyl 5-methyl-1-(pyridin-3-yl)-1*H*-1,2,3-triazole-4-carboxylate (**L2**) were prepared according to standard methods reported in the literature [34], generating the precursor ester compounds. The esters were saponified with a solution of NaOH in order to generate the corresponding carboxylate sodium salts—compounds **L1** and **L2**, respectively.

**L1**: Yield: quant. IR (KBr, cm^−1^ ); ν: 3062, 3036 (CAr-H); 2981 (Csp_3_-H); 1630 (C=O-), 1508 (N=N); 1483, 1466, 1447 (CAr-CAr); 1446 (COO-as). ^1^H-NMR (500 MHz, DMSO) δ (ppm): 8.72 (s, 1H, H5), 8.71 (d, *J* = 1.3 Hz, 1H, H3), 8.02 (d, *J* = 7.2 Hz, 1H, H1), 7.66 (dd, *J* = 7.9, 4.6 Hz, 1H, H2), 2.45 (s, 3H, H12). ^13^C-NMR (500 MHz, DMSO) δ (ppm): 166.20 (C13), 151.13 (C3), 146.15 (C5), 144.32 (C8), 136.67 (C9), 134.12 (C1), 133.65 (C6), 125.64 (C2), 10.20 (C12). HR-ESI-MS for C_9_H_7_N_4_O_2_Na [M + H]^+^: calculated = 226.2, found = 226.1 (1 ppm).

**L2**: Yield: quant. IR (KBr, cm^−1^ ); ν: 3102, 3055 (CAr-H); 2981 (Csp3-H); 1606 (C=O); 1489 (N=N); 1473, 1446, 1421 (CAr-CAr); 1427 (COO-as). ^1^H-NMR (500 MHz, DMSO) δ (ppm): 8.77 (dd, *J* = 4.6, 1.5 Hz, 2H, H2, H4), 7.67 (dd, *J* = 4.6, 1.5 Hz, 2H, H1, H5), 2.57 (s, 3H, H12). ^13^C-NMR (500 MHz, DMSO) δ (ppm): 166.11 (C13), 151.97 (C2, C4), 144.46 (C6), 143.61 (C8), 136.20 (C9), 119.54 (C1, C5), 10.40 (C12). HR-ESI-MS for C_9_H_7_N_4_O_2_Na [M + H]^+^: calculated = 226.2, found = 226.0 (2 ppm).

#### 2.3.2. Synthesis of Tetraaqua–bis(5-methyl-1-(3-pyridin)-1*H*-1,2,3-triazole-carboxilate) Cadmium(II) [Cd(L1)_2_·4H_2_O] (**1**)

A solution of **L1** (10 mg, 0.0442 mmol) in H_2_O (5 mL) was added to a solution of Cd(NO_3_)_2_⋅4H_2_O (6.28 mg, 0.0221 mmol) in *n*-butanol (5 mL). The resulting clear solution was kept at room temperature for 30 days until the crystals formed. The colorless crystals of compound **1** were filtered, washed with dimethylformamide (DMF), and dried in air. The yield was 0.0084 g (70%).

#### 2.3.3. Synthesis of Catena-tetra(5-methyl-1-(4-pyridin)-1*H*-1,2,3-triazole-carboxilate) Cadmium(II) Pentahydrate [Cd(L2)_4_]_n_ (**2**)

A solution of **L2** (10 mg, 0.0442 mmol) in H_2_O (5 mL) was added a solution of Cd(NO_3_)_2_⋅4H_2_O (6.28 mg, 0.0221 mmol) in ethanol (5 mL). The mixture was homogenized in an ultrasonic bath at 60 °C for 40 min, and then placed in a Teflon-lined stainless steel vessel, heated to 120 °C for three days, and cooled to room temperature over 24 h. Colorless needle crystals of compound **2** were obtained. The yield was 0.0063 g (60%). 

## 3. Results

### 3.1. Syntheses of ***L1*** and ***L2***

**Scheme 1:**
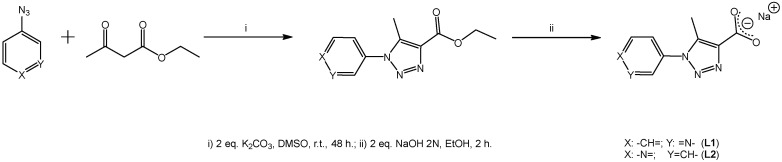
General synthetic route of ligand **L1** and **L2**.

The syntheses of both compounds were carried out according to the literature [34,35], using [3 + 2] dipolar cycloaddition between *n*-pyridyl azide (*n* = 3 or 4) and a 1,3-dicarbonyl compound such as ethyl acetoacetate (see Scheme 1). In general, the yields of the ester precursors are in the range of 35-70% and the yields of the sodium carboxylate salts **L1** and **L2** are quantitative.

The obtained compounds were hygroscopic, and consequently had to be stored in a dry box or under an in inert gas atmosphere. On the other hand, the ligand **L1** and **L2** are stable under atmospheric conditions. Both are white powders that are soluble in water and other protonic solvents, such ethanol or hot methanol.

Finally, suitable single crystals for XRD analysis were isolated. The crystal structure data reveal that both compounds crystallize in their protonic forms (see Section 3.2)

### 3.2. Syntheses of ***1*** and ***2***

**Scheme 2:**
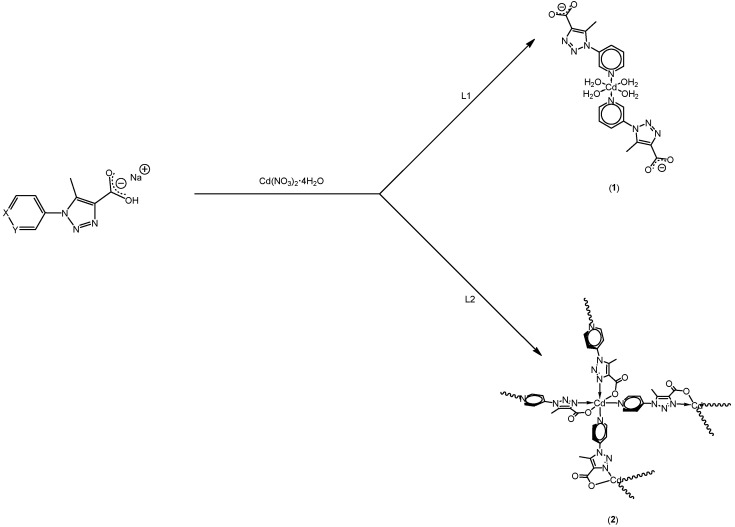
General synthetic route of compounds **1** and **2**.

The Cd(II) compounds were obtained using immiscible liquid for ion diffusion, generating single crystals in the interphase. In the case of [Cd(L1)_2_·4H_2_O] (**1**), the single crystals were manually isolated out of the reaction mixture of Cd(NO_3_)_2_·4H_2_O and **L1** in a mixture of 1:1 H_2_O and *n*-butanol. In the same way, the synthesis of [C_18_H_14_CdN_8_O_4_]_n_ compound **2**, gave rise to suitable crystals from the reaction mixture of Cd(NO_3_)_2_·4H_2_O and **L2** in a mixture of 1:1 H_2_O/ethanol. The stoichiometric ratio between Cd(II) salt and **L1** or **L2** was 1:2, respectively (see Section 2.3 for more details). Both compounds generated clear colorless single crystals for XRD analyses. In particular, for compound **2**, it was synthesized via solvothermal methods. According to the structural nature of these ligands, compound **1** generated a discrete coordination compound. Meanwhile, compound **2** generated a coordination polymer’s 3D architecture (see Scheme 2 and Section 3.3 for more details.).

### 3.3. Crystallographic Studies

**Figure 1:**
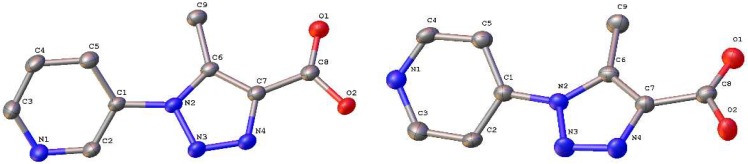
ORTEP plot for compounds **L1H** (**left**) and **L2H** (**right**). Hydrogen atoms were omitted for clarity’s sake. Thermal ellipsoids were drawn with 30% of probability.

The crystal structures and chemical compositions of all compounds were established by the single-crystal X-ray diffraction method. The molecular structures of **L1** and **L2** show their protonated forms **L1H** and **L2H** (see Figure 1), corresponding to their respective carboxylic acids. **L1H** crystallizes in the orthorhombic system with space group P*na*2_1_, and **L2H** crystallizes in a monoclinic system with space group *Cc*, both compounds with four molecular entities per unit cell with non-centrosymmetric settings. All the distances and angles are normal. The bond lengths between single and double bonds are typical for these types of compounds [36]. It can observed that there is a loss of coplanarity between the respective heterocycles (*n*-pyridyl and 1,4-disubstituted-1,2,3-1*H*-triazole moieties), where the torsion angle between their heterocycles is lower in **L2H** than in **L1H** with 36.8(5) and 39.1(8)°, respectively, following the same tendency in similar compounds previously reported with the same moieties [34,37].

**Figure 2:**
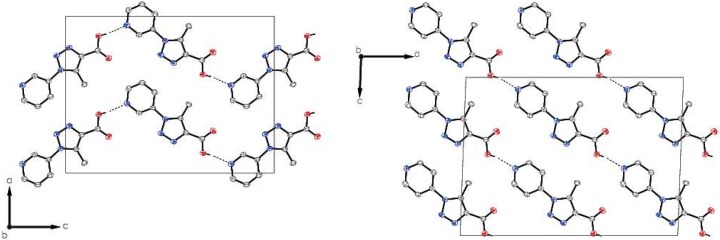
Crystal packing of compound **L1H** (**left**) and **L2H** (**right**).

The crystal structures of both compounds show hydrogen bonding interactions generated with -O(2)-H(2)···N(1), generating slabs along the (101) plane with graph set $C_{1}^{1}\left(9\right)$ in the crystal packing (see Figure 2). This situation has been reported before in compounds with similar features [37,38].

**Figure 3:**
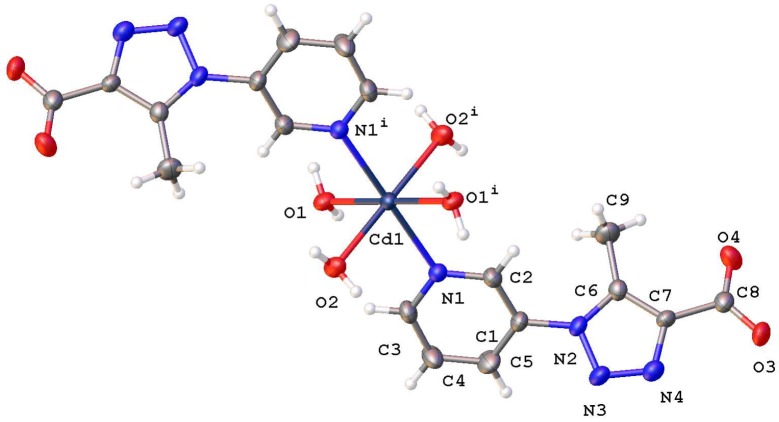
View of local coordination geometry at the Cd(II) center in compound **1** (30% ellipsoid probability). Symmetry code (i) is 1 − x,1/2 + y,3/2 − z.

**Figure 4:**
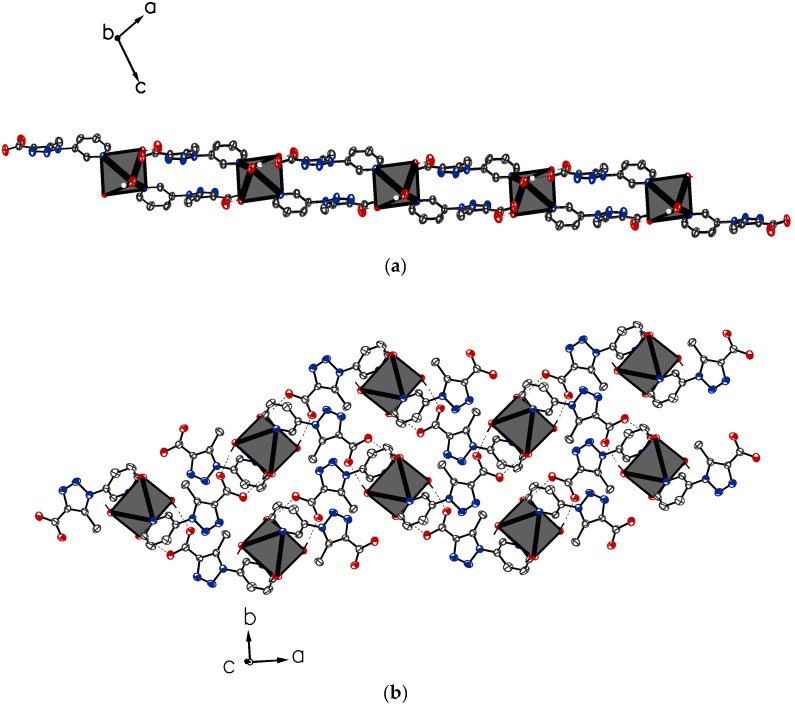
Slabs generated along the (101) plane (**a**) and the (110) plane (**b**), showing the coordination polyhedral.

In compound **1**, the asymmetric unit contains a Cd(II) cation, two water molecules, and two molecules of **L1**. The Cd(II) ion lies on an inversion center and is hexacoordinate with a [N_2_O_4_] coordination sphere (four water molecules and two N donor atoms from the pyridyl moiety of **L1**) in a distorted octahedral geometry (see Figure 3). The bond lengths and angles around the Cd(II) ion are in the range of 2.280(3)–2.294(3) Å for Cd-O, and 2.358(4) Å for Cd-N. The angles O-Cd-O and O-Cd-N are 86.57(13)-180.0° and 88.59(14)-91.41(14)°, respectively. These bond lengths and angles are similar to the reported values of related Cd(II) complexes containing carboxylate and pyridyl fragments [39,40]. Compound **1** exhibits parallel packing, generating a slab along the (101) plane (see Figure 4). Moreover, an induced hydrogen bond framework in the whole cell generates a spiral shape, due to the *2_1_*-screw axis and perpendicular glide plane. The hydrogen bonding interactions along the slab are located between water molecules in the equatorial positions of the coordination core and carboxylate fragments, specifically the O(3) and O(4) atoms.

The coordinated water molecules and the carboxylate groups are involved in the formation of a two-dimensional hydrogen-bonded network, which consolidates the crystal packing (see Figure 4).

**Figure 5:**
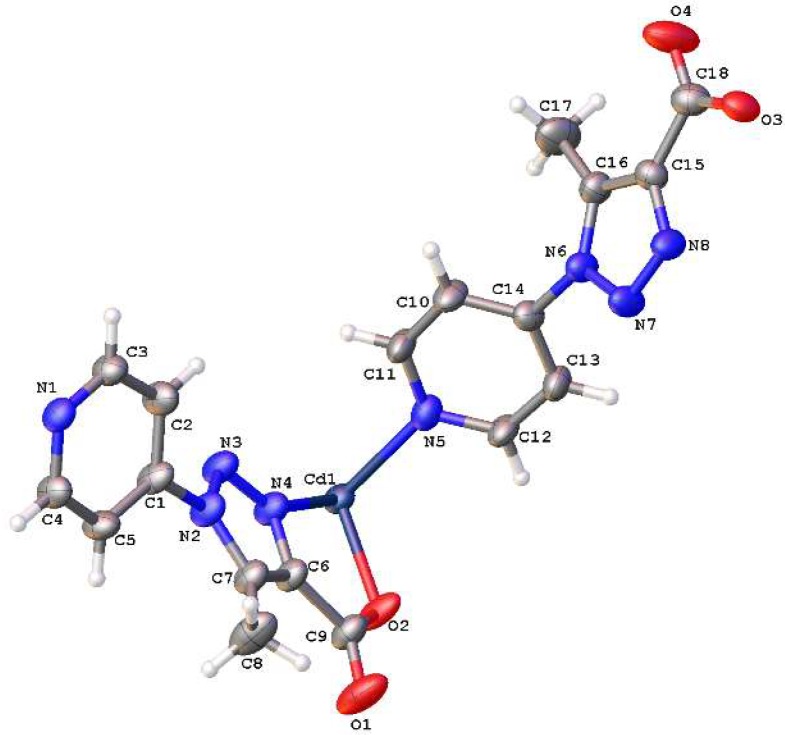
The asymmetric unit of compound **2**.

The crystal structure and chemical composition of compound **2** were established by the single-crystal X-ray diffraction method. The asymmetric unit of compound **2** contains a Cd(II) cation, the coordination environment of which consists of two N atoms of the pyridine, as well as triazole fragments and one O atom of the carboxylate group from two ethyl 5-methyl-1-(pyridin-4-yl)-1*H*-1,2,3-triazole-3-carboxylate ligands **L2** (Figure 5). In the symmetry-unique part of the molecule, the pyridine and chelate ring (N4/C6/C9/O2/Cd1) form a dihedral angle of 89.5(3).

**Figure 6:**
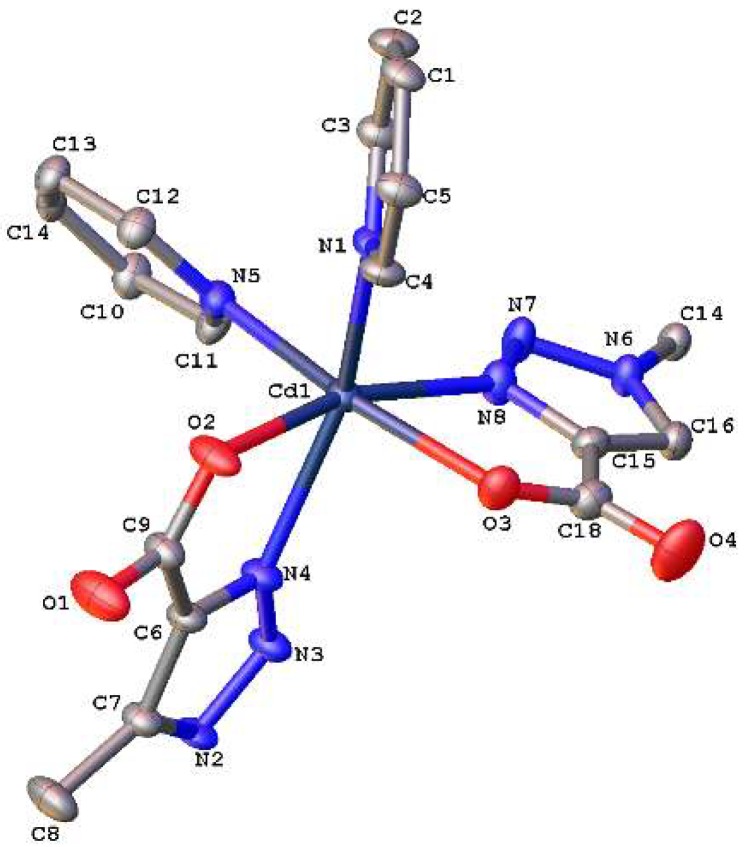
Coordination environment of [Cd(L2)]n cores.

The Cd(II) ion is coordinated in a slightly distorted octahedral geometry by four N atoms and two O atoms from the ethyl 5-methyl-1-(pyridin-4-yl)-1*H*-1,2,3-triazole-3-carboxylate ligands, (Figure 6). The Cd-O distances range from 2.278(3)–2.296(3) Å, and the Cd-N distances range from 2.291(3)–2.348(3) Å. The O-Cd-O angle is 94.64(11)°, and the O-Cd-N and N-Cd-N angles are in the range of 71.41(10)–166.39(10 and 89.38(11)−154.39(11)°, respectively. The Cd–O (carboxylate) and Cd–N bond parameters are in agreement with related Cd(II) complexes containing carboxylate and pyridyl fragments [39,40]. The mean plane defined by pyridine rings (N1/C1/C2/C3/C4/C5 and N5/C10/C11/C12/C13/C14 for plane 1 and plane 2, respectively) makes an angle of 123.81(14)°, and the chelate mean plane defined by Cd1/N8/C15/C18/O3 and Cd1/N4/C6/C9/O2 (plane 3 and plane 4, respectively) makes an angle of 89.00(10)°.

**Figure 7:**
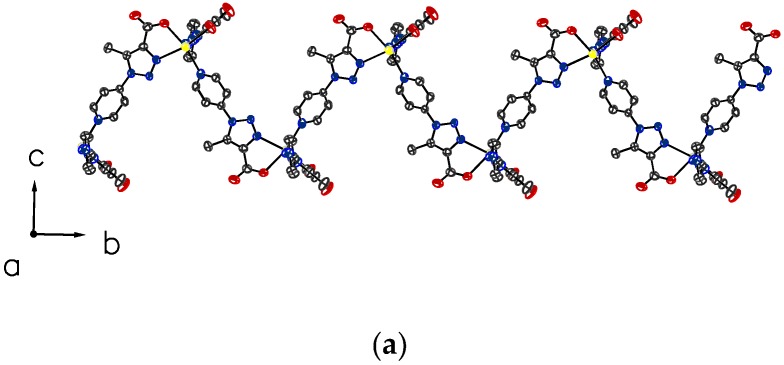

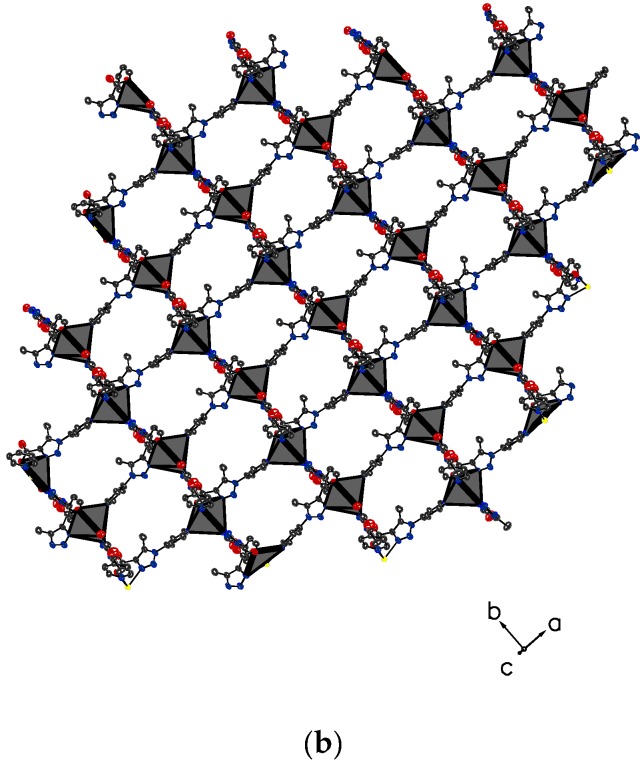
Three-dimensional (3-D) metalorganic frameworks along to plane (100) (**a**) and the plane (111) (**b**).

The main structural comparison between compounds **1** and **2** shows the isomeric positional effect in the ligand, because the ligands of compound **1** generate discrete molecular systems, while its positional isomer ligand 2, generates 3D metal–organic frameworks (see Figure 7).

**Figure 8:**
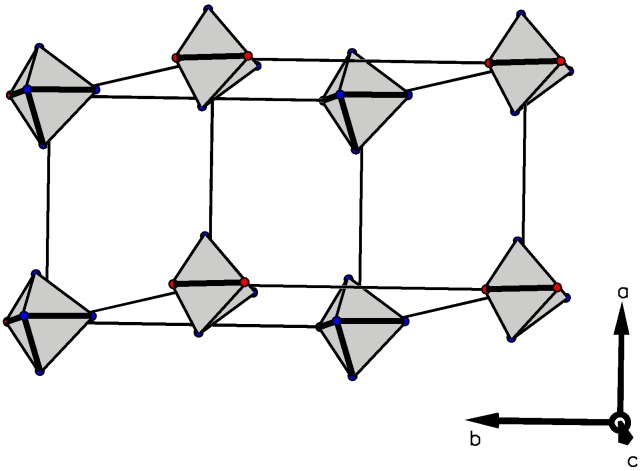
Supramolecular Boxes.

Another structural consequence of the isomeric effect is the generation of voids within the MOF material, which have a rhombohedral shape with a volume of 1337 Å^3^. These voids make this MOF a good candidate as a storage material or luminescent sensor, due to the ability to catch small molecules within in the voids (see Figure 8).

### 3.4. Thermal Stability Studies

**Figure 9:**
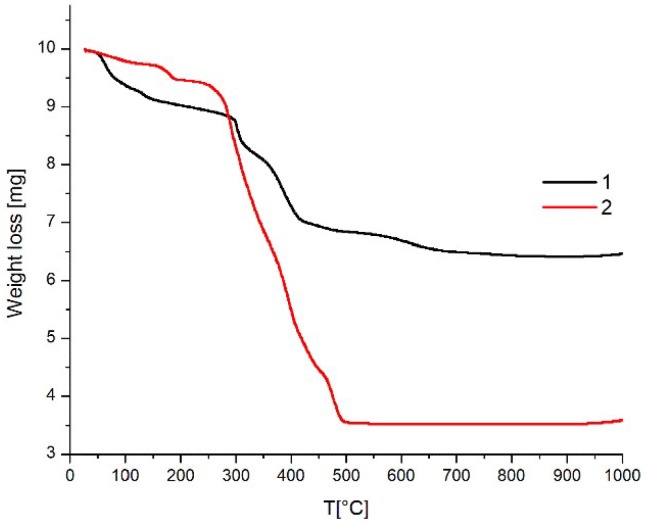
TGA plot of compounds **1** and **2**.

The TGA of both compounds were recorded with a heating rate of β = 10 °C·min^−1^ under a dynamic nitrogen atmosphere in the temperature interval of 20–1000 °C. All curves are shifted to a higher temperature at constant heating rate. The TG curves show a five-step weight loss until total decomposition. Compound **1** shows a decomposition starting at ca. 60 °C, with total decomposition over 600 °C. The first step corresponds to around two H_2_O molecules, due to moisture present in the sample (~6%). In the second step at ~150°C, the weight loss of water ligand molecule was ~3%. Over 300 °C, the decarboxylation from the ligand was founded (~8%). The two following steps correspond to progressive decomposition of the compound **1**. The TG curve of compound **2** shows a two-step decomposition curve. The first one at 186 °C represents a weight loss of 5% (two water molecules). The second step at ca. 290 °C corresponds to progressive decomposition of the organic ligand. The final compound after total decomposition of product **2** corresponds to cadmium oxide. (Figure 9).

### 3.5. Emission Spectra Measurements

**Figure 10:**
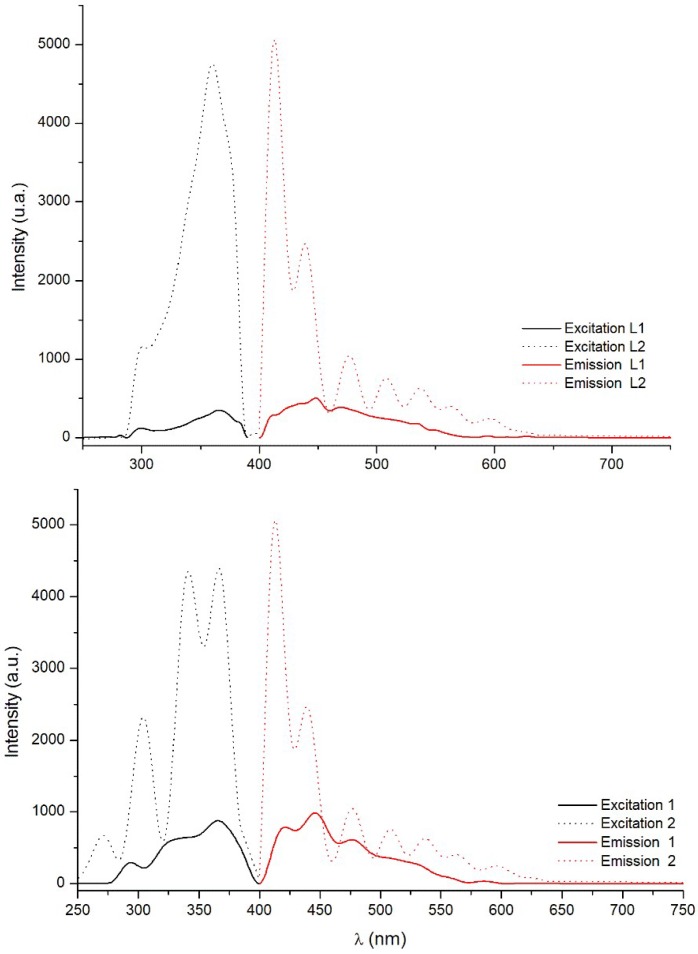
Deconvoluted excitation (black) and emission (red) spectra for compounds **L1** and **L2** (**top**) and compounds **1** and **2** (**bottom**).

The room temperature, solid-state excitation/emission deconvoluted spectra of each compound are shown in Figure 7, and their respective values in Table 2. These crystalline solids have interesting luminescent properties, with slight differences in their spectra (see Figure 10). There are no important differences between the spectra of the ligands with respect to the complexes, so it is possible to infer that the metal centers are not contributing to the molecular orbitals involved in the luminescence response.

In general, each compound shows the maximum excitation peaks at approximately 365 nm (see Table 2). The blueshift of the complex spectra may be attributed to the chelating or bridging effects of the ligands, due to their isomeric effect over the metal centers. Moreover, the bonding interaction between donor atoms and the Cd(II) center are slightly larger, agreeing with the Cambridge Crystallographic Data Base [41], which means that the contribution of the Cd(II) ion is negligible, explaining the slight blue-shifted bands, and focusing mainly on π–π* type transitions and the practically negligible metal-ligand charge transfer (MLCT) or ligand-metal charge transfer (LMCT), according to previously reported Cd(II) frameworks [42]

The spectra of all the compounds show important differences with respect to their luminescent intensities, which could be a consequence of the planar effect between pyridyl and triazole rings in the solid state. The compound **L1** could be less coplanar than compound **L2**; this difference is reflected in each complex, where the torsion angles between the fragments are 46.8(7)° for **L1** and 42.8(5)° for **L2**. Another plausible explanation for this difference could be due to the difference between the transition dipole moments of both compounds, where compound **L2** is higher than **L1**, as a consequence of a greater coplanar effect in compound **L2**.

For complexes **1** and **2**, the differences in the intensities could be due to the presence of water molecules in the tetraaquo complex **1**, because water molecules quench the basal state S_0_, changing the luminescent absorption energy into vibrational energy released by O–H vibration modes [43]. The amplified luminescent response in the Cd(II) compounds does not correspond to major contribution of the metal centers. Rather, it is a linear response according to the number of ligands that each compound contains. Compound **1** contains two ligand units, while compound **2** has more ligand units due to its polymeric constitution. At this moment, we are working in the computational studies of a series of coordination polymers using DFT methods. In this work, the topological studies are also included.

## 4. Conclusions

We synthesized and characterized new types of Cd(II) complexes prepared via crystallization 2:1 with 5-methyl-1-(pyridin-3-yl)-1*H*-1,2,3-triazole-3-carboxylate (**L1**) and ethyl 5-methyl-1-(pyridin-3-yl)-1*H*-1,2,3-triazole-4-carboxylate (**L2**). The positional isomeric effect in compounds **L1** and **L2** gave rise to different types of Cd(II) compounds, a mononuclear discrete complex (**1**), and a Cd(II) framework, using **L2** as a linker between metal centers (**2**). Thermal analyses of compounds **1** and **2** reveal that both compounds are stable over 200 °C, being good candidates for the preparation of luminescent materials.

## Figures and Tables

**Table 1:** Crystal data parameters for compounds **L1H**, **L2H**, **1**, and **2**.

Compound	L1H	L2H	1	2
Empirical Formula	C_9_H_8_N_4_O_2_	C_9_H_8_N_4_O_2_	C_18_H_22_CdN_8_O_8_	[C_18_H_14_CdN_8_O_4_]_n_
Formula mass, g·mol^−1^	204.19	204.19	590.84	518.77
Collection T, K	295.21	296.27	295.51	297.22
crystal system	orthorhombic	Monoclinic	Monoclinic	Monoclinic
space group	Pna2_1_	Cc	P2_1_/c	P2_1_/n
*a* (Å)	13.4617(12)	18.5248(10)	9.9864(5)	10.7368(8)
*b* (Å)	3.7613(3)	3.7425(2)	7.3650(4)	14.8147(14)
*c* (Å)	17.9448(16)	13.0186(7)	15.7758(9)	15.7859(14)
*α* (°)	90	90	90	90
*β* (°)	90	92.154(4)	104.846(3)	99.017(3)
*γ* (°)	90	90	90	90
*V* (Å^3^)	908.61(14)	901.93(8)	1121.57(11)	2479.9(4)
*Z*	4	4	2	4
ρ_calcd_ (g·cm^−^^3^)	14.926	1.504	1.750	1.389
Crystal size (mm)	0.402 × 0.24 × 0.108	0.414 × 0.129 × 0.114	0.13 × 0.102 × 0.073	0.459 × 0.433 × 0.258
*F*(000)	425.5	424.0	596.0	1032.0
abs coeff (mm^−1^ )	0.931	0.938	8.372	0.917
*θ* range (°)	9.86 to 122.94	9.556 to 117.866	9.16 to 118.36	5.5 to 56.75
range h,k,l	−14/15, −4/4, −19/19	−20/20, −4/4, −13/14	−11/11, −8/8, −17/17	−12/14, −1 9/19, −21/21
No. total refl.	13089	8282	24642	52471
No. unique refl.	1328	1242	1598	6169
Comp. *θ*_max_ (%)	94.0	97.0	98.3	99.4
Max/min transmission	0.765/0.904	0.865/0.899	0.413/0.543	0.663/0.78
Data/Restraints/Parameters	1328/5/138	1242/2/139	1598/0/164	6169/0/282
Final R [*I* > 2*σ*(*I*)]	R_1_ = 0.0490, wR_2_ = 0.1363	R_1_ = 0.0445, wR_2_ = 0.1004	R_1_ = 0.0392, wR_2_ = 0.0779	R_1_ = 0.0415, wR_2_ = 0.0922
R indices (all data)	R_1_ = 0.0618, wR_2_ = 0.1415	R_1_ = 0.0557, wR_2_ = 0.1077	R_1_ = 0.0614, wR_2_ = 0.0849	R_1_ = 0.0631, wR_2_ = 0.1041
Goodness of fit/F^2^	1.094	1.124	1.065	1.036
Largest diff. Peak/hole(eÅ^−3^)	0.33/−0.35	0.18/−0.15	0.72/−0.58	1.93/−0.63
Flack Parameter	0.0(5)	0.4(2)	---	---

**Table 2:** Excitation and emission data for compounds **L1**, **L2**, **1**, and **2**.

Compound	λ_max_ Excitation (nm)	λ_max_ Emission (nm)
**L1**	366	448
**L2**	360	413
**1**	366	446
**2**	366	412

## Supplementary Materials

The following are available online.
